# Genetic diversity and multiple introductions of porcine reproductive and respiratory syndrome viruses in Thailand

**DOI:** 10.1186/1743-422X-8-164

**Published:** 2011-04-12

**Authors:** Hein M Tun, Mang Shi, Charles LY Wong, Suparlark NN Ayudhya, Alongkorn Amonsin, Roongroje Thanawonguwech, Frederick CC Leung

**Affiliations:** 1 School of Biological Sciences, University of Hong Kong, Hong Kong SAR, China; 2 Emerging and Re-emerging Diseases in Animals, Research Unit, Faculty of Veterinary Science, Chulalongkorn University, Bangkok 10330, Thailand

## Abstract

Porcine reproductive and respiratory syndrome virus (PRRSV) is prevalent in Thailand, causing a huge impact on the country's swine industry. Yet the diversity and origin of these Thai PRRSVs remained vague. In this context, we collected all the Thai PRRSV sequences described earlier and incorporated them into the global diversity. The results indicated that PRRSVs in Thailand were originated from multiple introductions involving both Type 1 and Type 2 PRRSVs. Many of the introductions were followed by extensive geographic expansion, causing regional co-circulation of diverse PRRSV variants in three major pig-producing provinces. Based on these results, we suggest (1) to avoid blind vaccination and to apply vaccines tailor-made for target diversity, (2) to monitor pig importation and transportation, and (3) to implement a better biosecurity to reduce horizontal transmissions as three potentially effective strategies of controlling PRRS in Thailand.

## 1. Introduction

PRRSV is a major swine disease virus causing economic losses in swine industry worldwide including Thailand. This disease was first reported during almost concurrent epidemics in the North American countries (late 1980s) [1,2] and in the European countries (early 1990) [3]. The causative agent, porcine reproductive and respiratory syndrome virus (PRRSV) belongs to the family Arteriviridae in the order Nidovirales [4]. It is an enveloped virus containing a positive-sense RNA genome of approximately 15 kb in length, encoding at least nine open reading frames (ORFs) including ORF1a, 1b, 2a, 2b, and 3-7 [5]. Among them, ORF5 encoding the major envelope protein is often used for phylogenetic analysis because of its high variability. PRRSV can be divided into two major genotypes: Type 1 (European strains) and Type 2 (North American strains). Both genotypes are found to be genetically and antigenically heterogeneous [6,7]. According to the recent reports for PRRSV classification, Type 1 PRRSV is divided into 3 subtypes. Among them, the cosmopolitan Subtype I was further divided into 12 different clades [8,9]. For Type 2 PRRSV, 9 well-defined lineages have been described [8,10].

PRRSV has been circulating in Thailand for a long time. Seropositive animals could be traced back to as early as 1989, and seropositive rate increased annually from 1991 to 2002 [11]. In 1996, the first Thai PRRSV was isolated and was identified as Type 2 PRRSV [12]. A few years later, Type 1 PRRSV was also reported in Thailand, and some was found to be co-circulating with Type 2 PRRSV within the same herd [11]. Despite of the effort on characterizing PRRSV diversity in Thailand, the origin and epidemiological history of the viruses remain unknown. This study re-analyzes previously characterized Thai sequences in the context of global PRRSV diversity, which helps shed lights on the potential origin and prevalence of different variants of PRRSVs in Thailand.

## 2. Materials and Methods

### 2.1 Viral isolation

All Thai PRRS viruses were isolated from nursery pigs (3-8 week of age) showing sign of PRDC in different regions of Thailand during 2000 to 2008. All pig farms are multi-site farms and practicing partial all in-all out and mostly continuous-flow system. The infected pigs or tissue samples or sera were submitted to the Chulalongkorn University-Veterinary Diagnosis Laboratory (CU-VDL). Virus isolation was performed by using MARC-145 cells and PRRSV positive result was confirmed by PCR and Immunoperoxidase monolayer assay (IPMA) using SDOW17 [11,13].

### 2.2 Data sets

Four hundred and twenty two complete Type 1 and five hundred and eighty one complete Type 2 PRRSV ORF5 gene sequences were downloaded from Gene Bank and PRRSV database http://prrsvdb.org/ as reference backbone. The sequences are comprised of sequences representative of diversity of Type 1 [8] and Type 2 [10] and all available Thai PRRSV ORF5 sequences (Table 1). All Thai PRRSVs were sequenced at CU-VDL during 2000 to 2008 [11,13].

**Table 1:** Thailand PRRSVs analyzed in this study

Virus ID	Location	Year of isolation	Genotype	Accession number
01CB1*	Chonburi	2001	Type 1	AY297119
01RB1	Ratchaburi	2001	Type 1	AY297120
02BR1	Burirum	2002	Type 1	AY297121
02CB12	Chonburi	2002	Type 1	AY297122
02SB2	Saraburi	2002	Type 1	AY297123
02SB3	Saraburi	2002	Type 1	FJ908074
03RB1	Ratchaburi	2003	Type 1	AY297124
08RB103	Ratchaburi	2008	Type 1	FJ908075
08NP144	Nakhon Pathom	2008	Type 1	FJ908076
00CS1	Chachoengsao	2000	Type 2	AY297111
01NP1	Nakhon Pathom	2001	Type 2	AY297112
01NP1.2*	Nakhon Pathom	2001	Type 2	DQ056373
01UD6	Udorn Thani	2001	Type 2	AY297113
02CB13	Chonburi	2002	Type 2	AY297114
02KK1	Khonkhen	2002	Type 2	AY297115
02PB1	Prachinburi	2002	Type 2	AY297116
02SP2	Suphanburi	2002	Type 2	AY297117
02SP3	Suphanburi	2002	Type 2	AY297118
07NP4	Nakhon Pathom	2007	Type 2	FJ908077
08NP147	Nakhon Pathom	2008	Type 2	FJ908078
08NP148	Nakhon Pathom	2008	Type 2	FJ908079
08RB51	Ratchaburi	2008	Type 2	FJ908080
08RB154	Ratchaburi	2008	Type 2	FJ908081
08RB160	Ratchaburi	2008	Type 2	FJ908082

* Thai PRRSV prototypes.

### 2.3 Phylogenetic reconstruction

The ORF5 nucleotide sequences were aligned in MUSCLE v3.6 [14], using default settings with minor manual adjustments. The alignments were then screened for recombination using Recombination Detection Program v.2 [15]. No recombination was found in the alignment. Then, a phylogeny was constructed using a Bayesian Markov Chain Monte Carlo (BMCMC) method implemented in MrBayes v.3.2 [16]. A general time-reversible nucleotide substitution model with 4 categories of gamma-distributed rate heterogeneity and a proportion of invariant sites (GTR+ Γ4+I) were used. The posterior distribution of trees and model parameters were summarized from Markov Chain Monte Carlo sampling over 10 million generations, during which trees were sampled every 1000 generations. We adopted the classification system from the previous report [8,10] for lineage diversity of Thai sequences. Average pairwise genetic distances between two lineages were calculated to evaluate the interlineage genetic diversity.

## 3. Results

### 3.1 Type 1 PRRSV in Thailand

**Figure 1:**
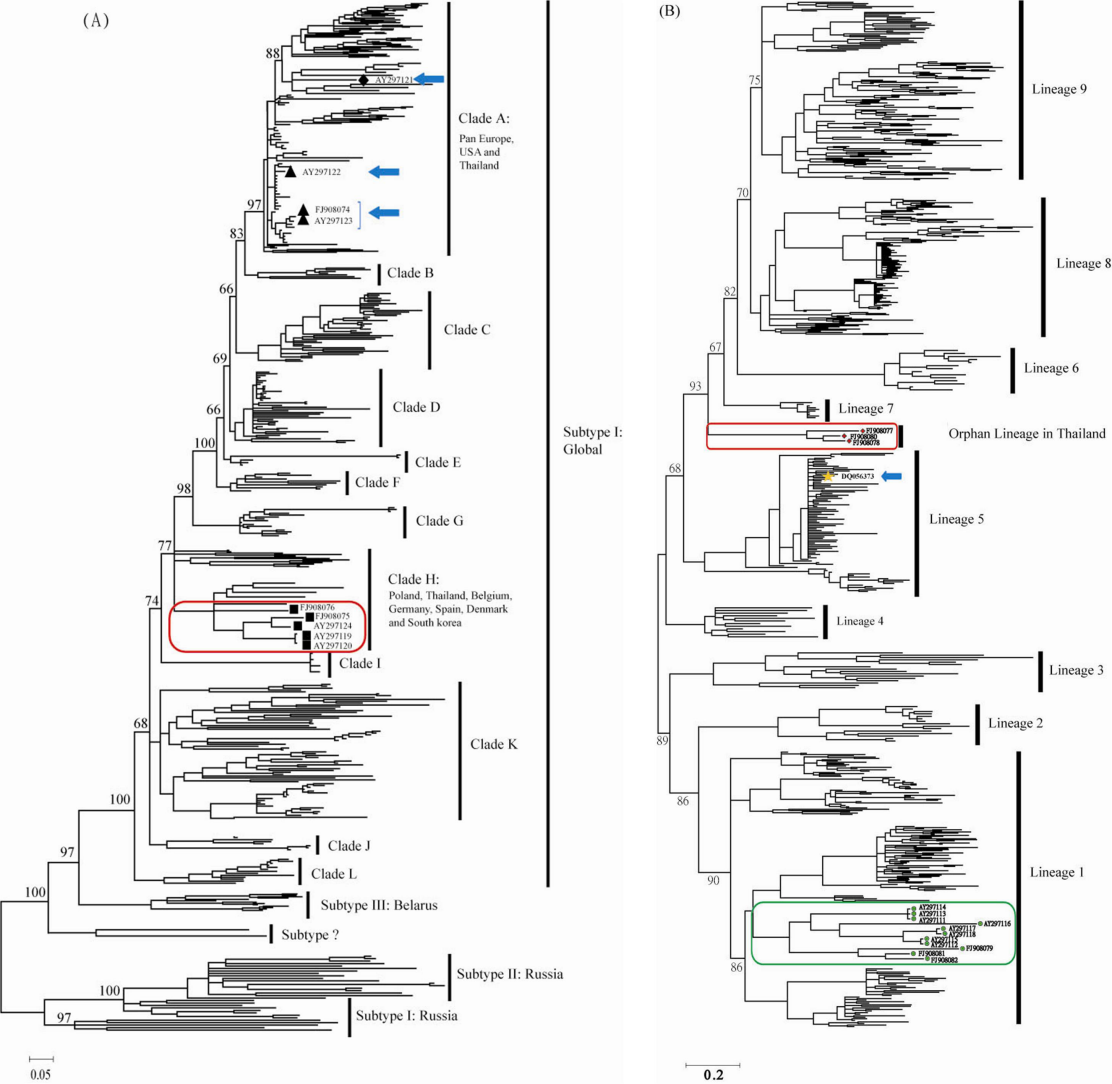
**Lineage identification of multiple introductions of Thai PRRSVs**. The phylogenetic trees were constructed in MrBayes and were mid-point rooted. For clarity, only the bootstrap values of selected and well-supported nodes were shown. (A) Thai Type 1 PRRSVs and (B) Thai Type 2 PRRSV on global context show multiple introductions. Blue arrows and colored rectangles show different lineages of Thai PRRSVs.

Phylogenetic analysis indicated that there were at least four independent introductions of Type 1 PRRSV into Thailand during 2000 to 2008. Among these introductions, three of them were from Clade A (Lelystad-like) while the other from Clade H. The definite sources of these introductions are difficult to identify due to complex geographic constituents for both clades (Figure 1A). Nevertheless, one Thai sequence (02CB12, 2002) was found to be closely related to the prototype (LV) with 98.5% of similarity as well as Porcilis^® ^PRRS with 99.1% of similarity (Intervet, The Netherlands) in Clade A (Figure 1A) indicating virus sample 02CB12 is probably a vaccine re-isolate or a vaccine descendant. Despite multiple introductions, the prevalence and diversity of Type 1 PRRSV in Thailand was quite limited except for those within Clade H (n = 6), which had an average genetic distance of 7% and a maximum pair of genetic distance of 10%.

### 3.2 Type 2 PRRSV in Thailand

Type 2 PRRSV in Thailand was originated from at least three independent introductions, which is evidenced by three monophyletic clusters: one (n = 9) within Lineage 1 while another one (n = 1) within Lineage 5 and the other (n = 3) as an orphan cluster that is beyond the description of current classification system by Shi et al (2010b). The phylogenetic backbone suggested this orphan cluster had closer relation to Lineage 6-9 forming a monophyl supported by high posterior probability (0.93). Nevertheless, further classification for this cluster remains unattainable. Since this orphan cluster had larger than 10% average genetic distances with those established lineages, we suggest this cluster as an independent lineage. Moreover, Thai prototype of Type 2 PRRSV (01NP1.2) has 100% nucleotide identity with MLV vaccine in Lineage 5 indicating an apparent vaccine re-isolate or vaccine descendant.

The major proportion of Thai Type 2 PRRSVs formed a single cluster within Lineage 1 (Figure 1B). This cluster had the largest diversity comparing to other Thai clusters, with an average genetic distance of 9% and a maximum pair of genetic distance of 14%. In addition, our estimation of the tMRCA of this cluster suggested an introduction time of around early 1990s if not earlier (Tun, personal communication).

### 3.3 Geographic distribution of PRRSV in Thailand

**Figure 2:**
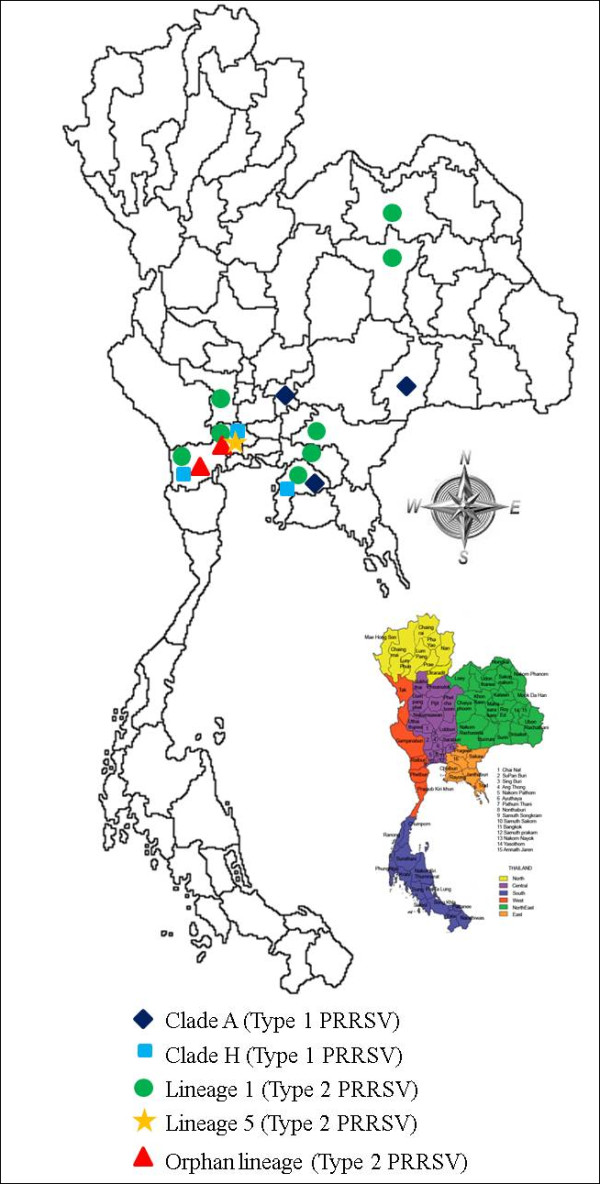
**Geographical lineage distributions of PRRSVs in Thailand**. Different colored shapes indicate independent introductions and extensive geographic expansion, causing regional co-circulation of diverse PRRSV variants in three major pig-producing provinces (Chonburi, Ratchaburi and Nakhon Pathom).

In order to examine the distribution spread of PRRSVs after their initial introduction into Thailand, geographical locations with different shapes were marked as previously described (in section 3.1 and 3.2) as Type 1 and 2 Thai clusters. Each represented an independent introduction event (Figure 2). For some provinces (Chonburi, Ratchaburi and Nakhon Pathom) in central Thailand, co-circulation of multiple clusters from the two types of PRRSV was observed whereas others had only one cluster of PRRSV. In addition, it is worth mentioning that the Thai cluster within Lineage 1 had the widest distribution, spreading to at least 8 provinces in Thailand (Figure 2).

## 4. Discussion

The appearance of the vaccine-related strains in Thailand is of interest since the Porcilis^® ^PRRS is not commercially available in Thailand. One possibility is the smuggling or unauthorized use of this modified live vaccine. Alternatively, it might originate from previous introduction of vaccinated breeder animals. In Thailand, most of the swine breeders and frozen semen have been continuously imported from both European and North American countries. Evidences of seropositivity have been reported in the imported pigs and semen from previous retrospective study [12]. Despite of the presence of vaccine-related PRRSVs in Thailand, their impact to the swine industry was most likely insignificant. It is because for each vaccine-related variant, only one sequence was identified in the phylogeny. And no transmission chain has been identified to be seeded by these vaccine strains..

Interestingly, three Type 2 PRRSV isolates collected from 2007 and 2008 belong to an orphan lineage which has limited diversity but is well isolated from any of the established lineages. This is also supported by NSP2 phylogeny, where the Thai orphan lineage (08NP147) is distantly related to other Type 2 PRRSV [17]. The missing diversity between the orphan lineage and other lineages are most likely due to incomplete sampling in Thailand. Indeed, the sampling in this study is limited by not only quantity, but also time span, compared to that of North America. Therefore, we suggest more intensive sampling and characterization of PRRSV in Thailand to fill in the diversity gaps.

Our recent biogeographic analyses on Type 2 PRRSV suggest that Lineage 6-9 were mostly endemic in the United States whereas Lineage 1 and 2 were originally circulating in Canada and later emerged in the United States due to intensive pig flow from Canada to the United States (Shi, unpublished data). Since the Thai orphan lineage is clustered within Lineage 6-9, it was most likely introduced from the United States. On the other hand, the Thai cluster within Lineage 1 was potentially introduced from Canada or United States. The Canadian origin of this cluster is more favored, because its common ancestor was estimated as early 1990s, which is earlier than the emergences of Lineage 1 and 2 PRRSV in the United States. Yet this perspective remains to be examined by incorporating earlier Canadian samples into the phylogenetic analyses.

Chonburi, Ratchaburi and Nakhon Pathom are three major Thai swine producing provinces located in the central region. Evidence based on our phylogenetic analyses show co-circulation of multiple clusters of both genotypes of PRRSV in these three provinces. This co-circulation of multiple PRRS viruses renders huge burden on the swine industry in these areas, because it might cause succession of PRRSV infection (due to lack of cross-protection for heterologous infection) and recombination. Since our analyses are based on ORF5 sequence alignment, no recombination events were detected but potential recombination breakpoint may exist in other part of the genome. In addition, we are able to identify a widely distributed Thai cluster (Lineage 1 of Type 2) originated from an introduction of approximately 20 years ago. Additionally, it is likely the movement of the PRRSV infected pigs between the herds that maintain the transmission chain of this particular cluster.

Our study demonstrates multiple introductions of PRRSV strains into Thailand, which result in co-circulation of diverse PRRSV strains in some areas. This posts difficulty on the vaccine selection since current monovalent vaccine has been shown to provide no or only partial protection against heterologous PRRSV strains [6,18,19]. Therefore, correct choice of vaccine is essential for prevention and control of PRRSV in Thailand. In addition to vaccination, the virus control measures should also aim for pig importation and transportation. First, an upgraded biosecurity system is required to prevent not only the introduction of new types of PRRSV into Thailand, but also the subsequent dissemination of the viruses among major pig-producing provinces. Second, it is necessary to establish a surveillance system which monitors the pig flows. This helps provide timely remedy in cases of outbreak.

## List of abbreviations

PRRSV: porcine reproductive and respiratory syndrome virus; ORF: open reading frame; PRDC: porcine respiratory disease complex; CU-VDL: Chulalongkorn University-Veterinary Diagnosis Laboratory; IPMA: Immunoperoxides monolayer assay; BMCMC,: Bayesian Markov Chain Monte Carlo

## Competing interests

The authors declare that they have no competing interests.

## Authors' contributions

HMT carried out the advanced phylogenetic studies and drafted manuscript. HMT, MS and CLYW participated in data analysis. SNNA did sampling and sequencing. AA and RT provided sequence data and helped to draft the manuscript. FCCL and HMT conceived the study, participated in its design and coordination. All authors read and approved the final manuscript.
